# Double Portal Vein: Report of a Rare Case

**DOI:** 10.7759/cureus.68835

**Published:** 2024-09-06

**Authors:** Feras A Almbaidin, Raed Jarrah, Mohammed Alduham, Jafar H Alrfou, Laith H Halasah

**Affiliations:** 1 Department of Hepatobiliary Surgery, Jordanian Royal Medical Services, Amman, JOR

**Keywords:** anatomical variation, azygous continuation of the ivc, double portal system, incidental finding, intestinal malrotation, left sided ivc, portal vein

## Abstract

This case report presents a rare incidental finding of a double portal system in a patient, discovered during an abdominal imaging study before undertaking surgical treatment for common bile duct (CBD) stones. The report provides a detailed account of the anatomical features of the double portal system observed in the patient. The clinical implications of this finding are significant, as it necessitates careful consideration during surgical planning or interventional radiology procedures to prevent inadvertent damage to the vascular structures. By discussing this case, the report aims to raise awareness among clinicians about the possibility of encountering such variations and the need for thorough preoperative imaging to ensure optimal patient outcomes.

## Introduction

The portal vein is a crucial component of the hepatic circulatory system, responsible for transporting nutrient-rich blood from the gastrointestinal tract to the liver. The portal vein also supplies around 30% of the hepatic arterial blood supply and around 70% of oxygenated blood to the liver [1]. Variations in portal vein anatomy are uncommon and can pose challenges during surgical procedures or liver interventions [2-5]. We present a case of an incidental finding of a double portal system, a rare anatomical variant.

## Case presentation

**Figure 1:**
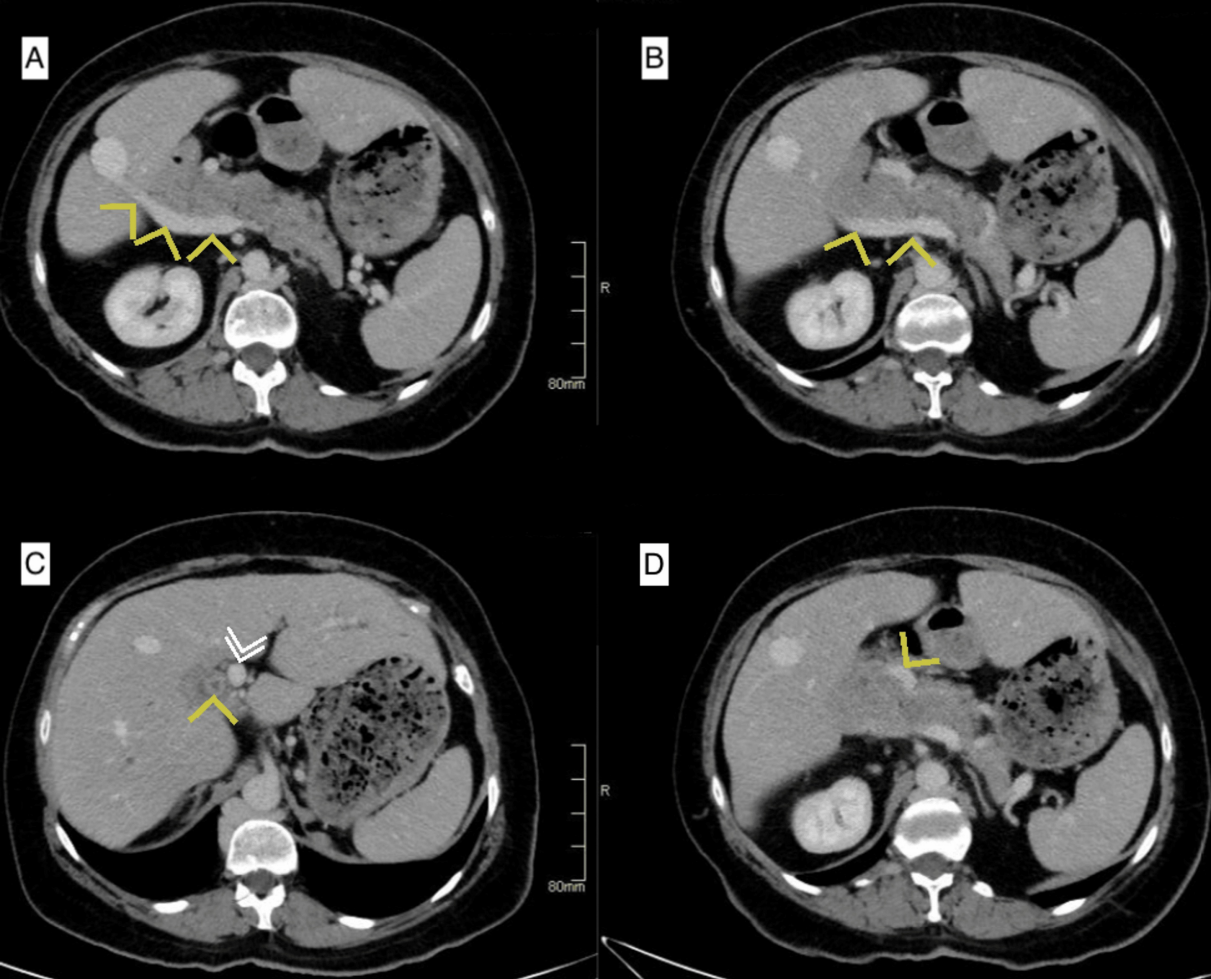
Computed tomography images showing the aberrant anatomy of the second portal vein (yellow arrows) traveling behind the pancreas and entering the liver near segment VI (A, B), a stone in the common bile duct (yellow arrow) next to the original portal vein (white double arrows) at the level of the porta hepatis (C), and the original portal vein (yellow arrow) coursing anterior to the pancreas (D).

A 60-year-old female, with a history of laparoscopic cholecystectomy in 1996, which was converted to an open approach due to bleeding from an unidentified vessel, was referred to our practice for surgical management of a common bile duct stone that was not amenable to endoscopic retrograde cholangiopancreatography (ERCP). An abdominal computed tomography (CT) scan was obtained at our facilities to better delineate the patient’s biliary anatomy. The CT scan revealed multiple incidental findings of intestinal malrotation, a left-sided inferior vena cava (IVC), azygous continuation of the IVC, and a double portal system, characterized by the presence of two distinct portal veins entering the liver. In this scan, the superior mesenteric vein was seen giving a branch anterior to the pancreas before joining the splenic vein behind the pancreas (Figure 1).

**Figure 2:**
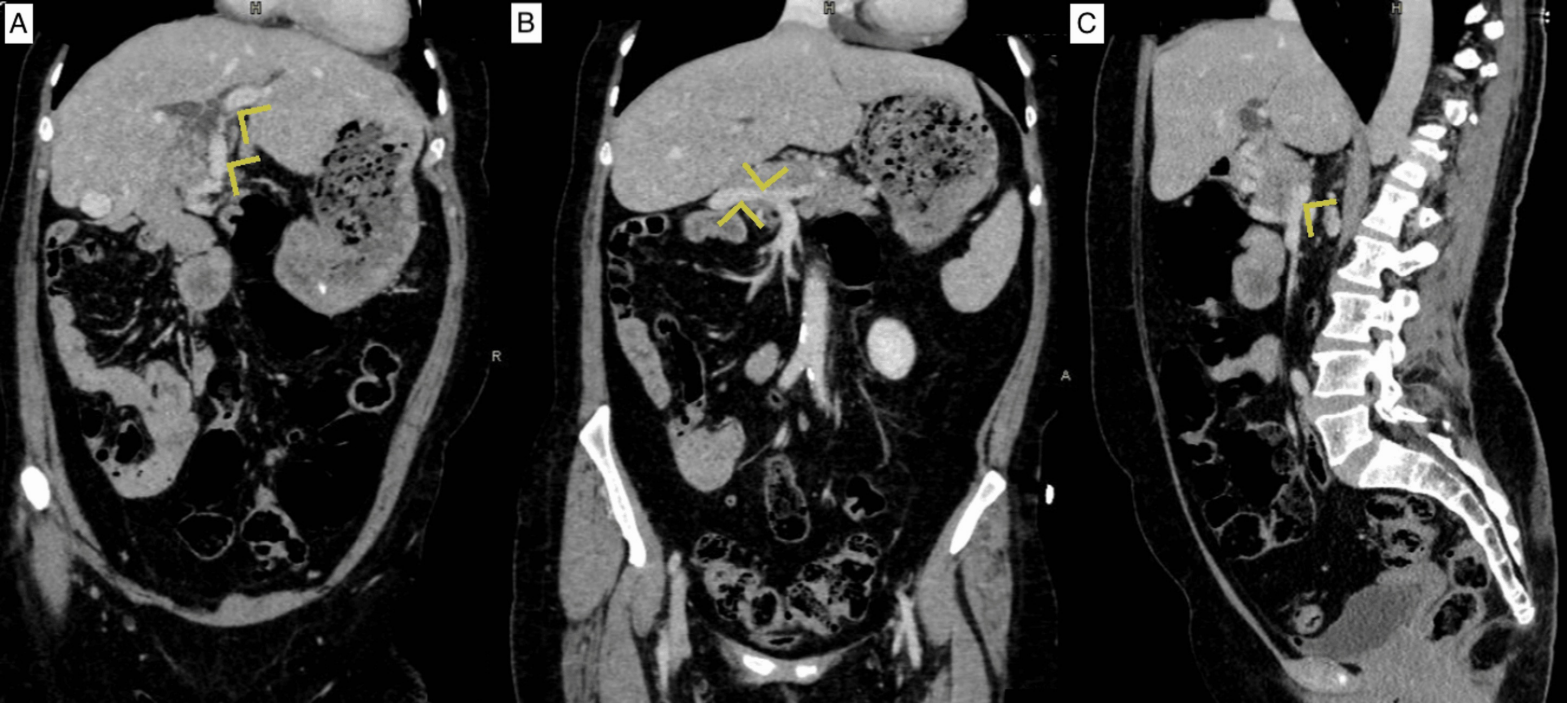
Computed tomography coronal and sagittal views showing the portal vein (yellow arrows) coursing anterior to the pancreas up to the porta hepatis (A), the second portal vein (yellow arrows) joined by the splenic vein posterior to the pancreas (B, C).

**Figure 3:**
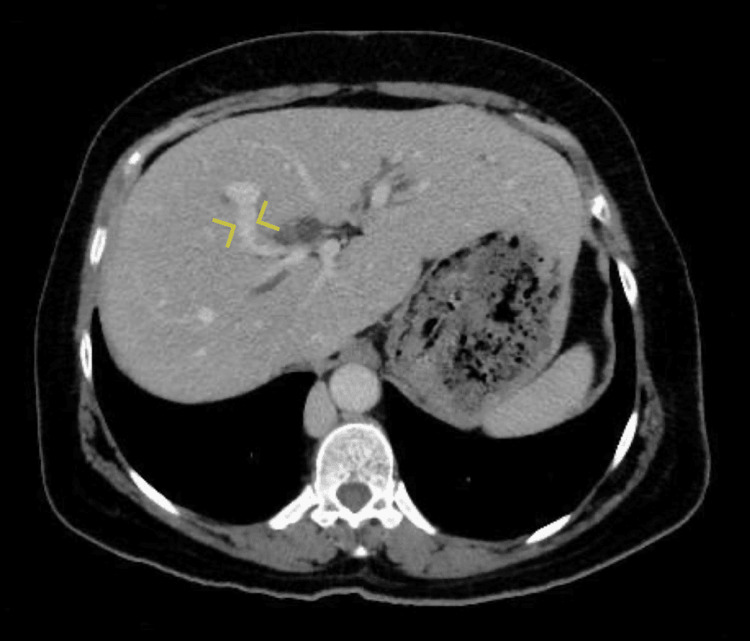
Communication (yellow arrows) between the two portal veins inside the liver parenchyma near segment VIII.

This anterior branch continues towards the porta hepatis as a portal vein, and the posterior branch joined by the splenic vein continues laterally to the right and enters the liver at its edge close to segment VI as the second portal vein (Figure 2). The two portal veins were noted to communicate inside the liver parenchyma near segment VIII (Figure 3).

**Figure 4:**
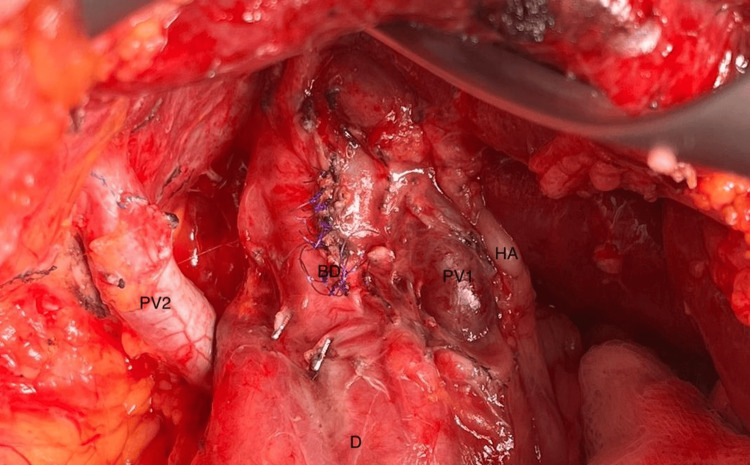
Intraoperative image showing the double portal anatomy; duodenum (D), hepatic artery (HA), common bile duct (BD), first portal vein going into the liver at its hilum (PV1), second portal vein going into the liver at segment VI (PV2).

Due to the patient’s previous surgical history, and her preference for an open common bile duct (CBD) exploration was performed, the patient’s anomalous anatomy was identified intraoperatively (Figure 4) behind dense adhesions from her previous operation. Other than meticulous dissection and care during the procedure these findings did not alter the surgical plan or the operation's course. Postoperatively the patient did very well and she was discharged home on postoperative day 3. 

## Discussion

The portal vein typically forms from the confluence of the superior mesenteric vein and the splenic vein posterior to the pancreatic head. It then divides into right and left branches at the porta hepatis, supplying the corresponding liver lobes [1]. The occurrence of a double portal system is a rare anatomical variation [2-4], often identified incidentally during radiological imaging or surgical procedures.

The etiology of a double portal system remains unclear and may be related to the embryonic development of the hepatic circulation. This variation could result from the persistence of two separate embryonic portal veins or an anomalous division of the portal vein during fetal development [2,3,5]. While anatomical variants of the portal vein can be present in up to 30% of patients [3], this patient's variant of the double portal vein, to the best of our knowledge, is quite rare in literature; furthermore, we are not able to find this anomaly in combination with intestinal malrotation in any other open source reports.

Clinical implications of a double portal system include potential challenges during liver surgeries, liver transplantation, and interventional radiological procedures involving the portal vein. Surgeons and interventional radiologists need to be aware of such anatomical variations to plan and execute procedures safely and effectively.

## Conclusions

The incidental finding of a double portal system is a rare anatomical variation that clinicians may encounter during abdominal imaging studies or surgical procedures. Awareness of such variations is essential for planning and executing safe and successful surgical and interventional procedures involving the hepatic circulatory system. Further research and anatomical studies are needed to better understand the prevalence, and clinical significance of double portal systems.
